# Identification of *Aedes aegypti* salivary gland proteins interacting with human immune receptor proteins

**DOI:** 10.1371/journal.pntd.0010743

**Published:** 2022-09-07

**Authors:** Edem Gavor, Yeu Khai Choong, Yonghao Liu, Julien Pompon, Eng Eong Ooi, Yu Keung Mok, Haiyan Liu, R Manjunatha Kini, J. Sivaraman

**Affiliations:** 1 Department of Biological Sciences, National University of Singapore, Singapore; 2 Infectious Diseases Translational Research Programme, Department of Microbiology and Immunology, Yong Loo Lin School of Medicine, National University of Singapore, Singapore; 3 Immunology Programme, Life Sciences Institute, Immunology Translational Research Program and Department of Microbiology and Immunology, Yong Loo Lin School of Medicine, National University of Singapore, Singapore; 4 Programme in Emerging Infectious Diseases, Duke-NUS Medical School, Singapore; 5 MIVEGEC, University of Montpellier, IRD, CNRS, Montpellier, France; 6 Department of Pharmacology, Yong Loo Lin School of Medicine, National University of Singapore, Singapore; National Institute of Allergy and Infectious Diseases, UNITED STATES

## Abstract

Mosquito saliva proteins modulate the human immune and hemostatic systems and control mosquito-borne pathogenic infections. One mechanism through which mosquito proteins may influence host immunity and hemostasis is their interactions with key human receptor proteins that may act as receptors for or coordinate attacks against invading pathogens. Here, using pull-down assays and proteomics-based mass spectrometry, we identified 11 *Ae*. *aegypti* salivary gland proteins (SGPs) (e.g., apyrase, *Ae*. *aegypti* venom allergen-1 [AaVA-1], neutrophil stimulating protein 1 [NeSt1], and D7 proteins), that interact with one or more of five human receptor proteins (cluster of differentiation 4 [CD4], CD14, CD86, dendritic cell-specific intercellular adhesion molecule-3-grabbing non-integrin [DC-SIGN], and Toll-like receptor 4 [TLR4]). We focused on CD4- and DC-SIGN-interacting proteins and confirmed that CD4 directly interacts with AaVA-1, D7, and NeST1 recombinant proteins and that AaVA-1 showed a moderate interaction with DC-SIGN using ELISA. Bacteria responsive protein 1 (AgBR1), an *Ae*. *aegypti* saliva protein reported to enhance ZIKV infection in humans but that was not identified in our pull-down assay moderately interacts with CD4 in the ELISA assay. Functionally, we showed that AaVA-1 and NeST1 proteins promoted activation of CD4^+^ T cells. We propose the possible impact of these interactions and effects on mosquito-borne viral infections such as dengue, Zika, and chikungunya viruses. Overall, this study provides key insight into the vector-host (protein-protein) interaction network and suggests roles for these interactions in mosquito-borne viral infections.

## Author summary

Here, we report our results from a pull-down assay and ELISA, which identified *Ae*. *aegypti* salivary gland proteins that interact with one or more of five human receptor proteins. Some of these interactions could affect the expression of costimulatory molecules involved in host defense against pathogens. This underscores the potential proviral or antiviral roles of these interactions on mosquito-borne viral infections. Our study provides a preliminary enquiry into the vector (mosquito)-host (human) interaction networks and how this interaction could be further investigated and harnessed as a strategy to augment existing vector-borne diseases control approaches.

## Introduction

As vectors, mosquitoes carry and transmit numerous pathogenic viruses, such as dengue (DENV), chikungunya (CHIKV) and Zika (ZIKV), as well as the deadly malaria parasites. Globally, over 700 million people are infected each year with mosquito-borne infections, resulting in over 1 million deaths annually [1]. Previous reports have shown the feasibility and potential of curbing mosquito-borne diseases by targeting mosquito saliva and midgut proteins [2–6].

Female mosquitoes infuse saliva to counter the host response of blood coagulation, platelet aggregation and vascular constriction and maintain uninterrupted blood acquisition. Unfortunately, infected mosquito inoculates viruses and its saliva into human hosts and contributes to virus transmission. The immunomodulatory, anti-inflammatory, and anti-hemostatic effects of saliva proteins enhance transmission of pathogens [3–5,7,8]. *Aedes aegypti* has over 120 salivary gland proteins [9–13] and several of these proteins have been shown to enhance viral and parasitic transmissibility and infectivity [4,5,8]. Some of these saliva proteins interact with human proteins to modulate the transmission and infection process [14–17].

For example, the mosquito proteins lymphotoxin ß Receptor inhibitor (LTRIN) and AaVA-1 interact with human proteins lymphotoxin ß Receptor (LTßR) and leucine rich pentatricopeptide repeat containing (LRPPRC), respectively. LTRIN-LTßR interactions abrogate LTßR dimerization and enhances ZIKV infection in humans [8], while AaVA-1-LRPPRC interaction promotes DENV and ZIKV infection in humans by activating autophagy in host immune cells [3]. The molecular interactions of saliva proteins with human protein receptors have not been fully documented. Such studies will help us understand the role of mosquito saliva proteins in viral transmission.

Some of the major players in host immunity against invading pathogens include the cluster of differentiation (CD) receptors, such as CD4 [14,18], CD14 [19] and CD86 [20,21], the dendritic cell-specific intercellular adhesion molecule-3-grabbing non-integrin (DC-SIGN) [22,23], and the Toll-like receptors (TLRs), including TLR-4 [24,25]. Some of these receptor proteins also serve as cellular entry receptors or attachment factors for arboviruses and enhance their transmission and infection.

Human CD4 is a major target for tick and mosquito saliva proteins such as the tick protein Salp15 and the mosquito protein SAAG-4 [16,26–28]. Salp15 directly interacts with the CD4 co-receptor on T cells, thereby inhibiting T cell signaling and facilitating *Borrelia burgdorferi* bacterial infection, the predominant cause of Lyme disease [14,27]. Moreover, *Ae*. *aegypti* SAAG-4, a novel salivary protein that induces IL-4, reprograms CD4+ T cells and suppresses the immune system [28]. However, there is no evidence whether this effect is dependent on CD-4-binding. DC-SIGN and CD14 receptor proteins function as attachment factors for many viruses, including DENV, ZIKV and CHIKV [22,23,29]. The crosstalk between CD86, CD80, CD103 and CD4 T cells provides a coordinated attack to clear invading pathogens [21]. A tick saliva protein, Japanin, reprograms DC responses to various stimuli *in vitro*; in particular, it changes the expression pattern of CD86 and promotes pathogen transmission [30]. However, similar to SAAG-4, there is no evidence whether this effect is dependent on DC-SIGN-binding. TLR4 is a significant target of several viruses [25]. For example, the DENV non-structural protein 1 (NS1) interacts with TLR4 on endothelial cells to induce the secretion and expression of macrophage migration inhibitory factors [31], thereby leading to NS1-induced vascular hyperpermeability in patients with DENV. Mammalian myeloid differentiation factor-2 (MD2) is a co-receptor required for TLR4 binding to lipopolysaccharides [32,33]. An MD2-like protein has been identified in *Ae*. *Aegypti*. Others show that silencing the *Anopheles gambiae* MD2-like family member, AgMDL1, significantly increases midgut *Plasmodium falciparum* infection levels, responsible for malaria disease [33,34].

Studying the complex interactions between human receptors and key mosquito saliva proteins will contribute to understanding molecular mechanisms of saliva-driven transmission enhancement that will aid in developing strategies for mosquito-borne disease control. Here, we conducted a pull-down assay using *Ae*. *Aegypti* salivary gland homogenates and five human receptor proteins as bait: CD4, CD14, CD86, DC-SIGN, and TLR4. We identified numerous key mosquito salivary gland proteins (SGPs) that interacted with one or more of these receptor proteins and confirmed their direct interaction using ELISA. We also showed the effect of some of these proteins on activation of CD4^+^ T cells. We propose and discuss the potential roles of these interactions on critical viral/parasitic infections.

## Results

### Extraction of *Ae*. *aegypti* salivary gland proteins (SGPs)

**Fig 1:**
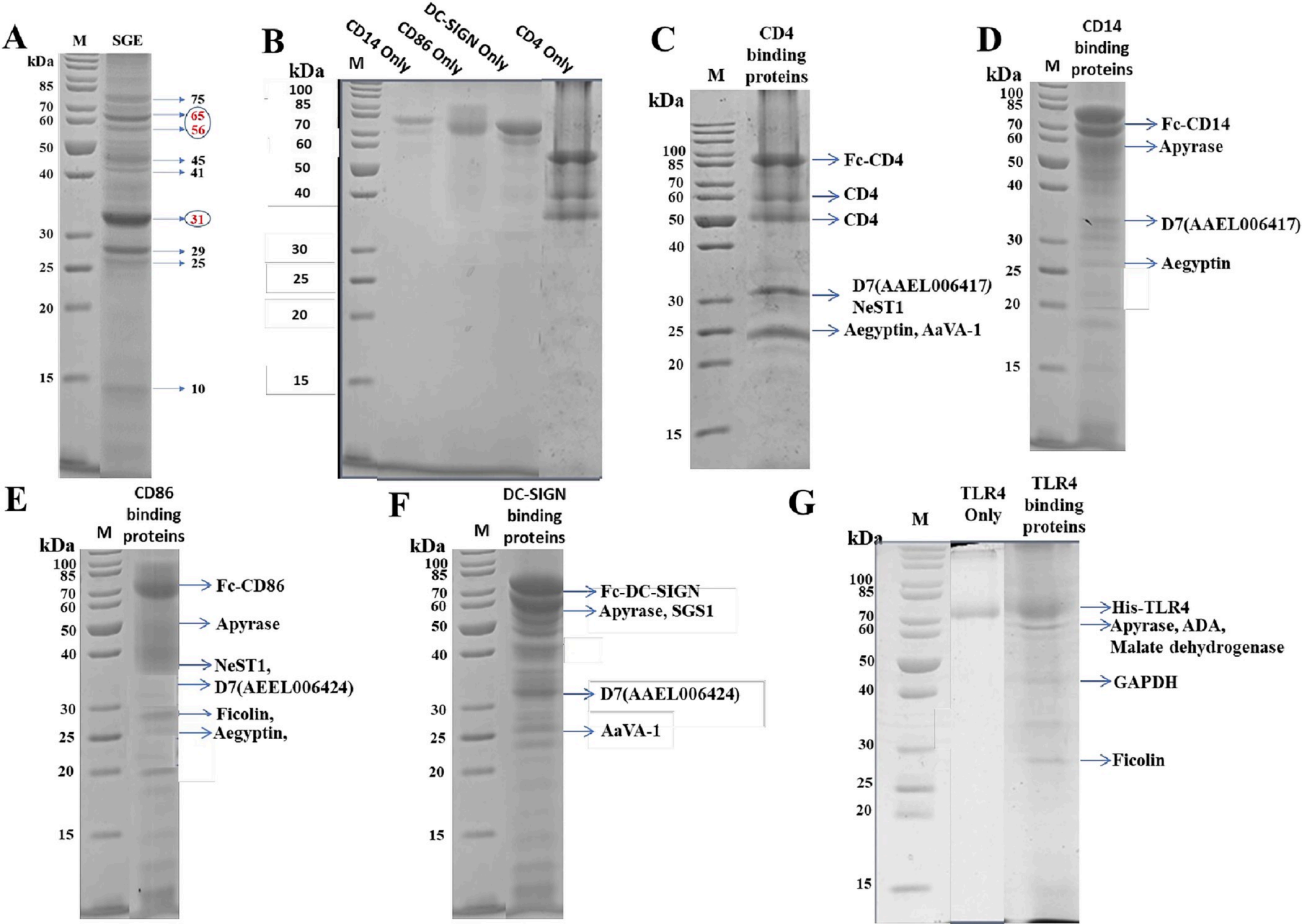
SDS-PAGE analysis of neat SGE, human receptor proteins and human receptor interacting SGPs. (**A**) *A*. *aegypti* SGE analysis showing 9 prominent bands and many smaller bands. The red font intense band labels with molecular weights of 56/65 kDa and 31 kDa, were previously analysed to contain seven and thirteen different proteins dominated by apyrase and D7(AEEL006424 and AEEL006417) proteins, respectively [42]. (**B**) CD14, CD86, DC-SIGN, and CD4 only control. (**C**) CD4-interacting SGPs. (**D**) CD14- interacting SGPs. (**E**) CD86-interacting SGPs. (**F**) DC-SIGN interacting SGPs. SGS1 is a 380 kDa protein. The identified lower molecular weight bands might be due to cleaved products as this protein is known to have three predicted cleavage sites [40]. (**G**) TLR4-interacting proteins. Due to post-translational modifications and similar molecular weight of SGPs, a single band could contain more than one protein merged into that band.

Over 2500 salivary glands were isolated from 2- to 3-day-old adult uninfected female *Ae*. *aegypti* mosquitoes. Female mosquitoes were used as only the female mosquitoes feed on blood and transmit pathogens. Uninfected mosquitoes were used to avoid contamination by virus particles in the salivary gland extract (SGE) that might interfere with the binding of salivary gland proteins (SGPs) to human receptor bait proteins. The total protein content from female mosquito SGEs was determined using a Bio-Rad Protein Assay (Bio-Rad), with the protein mixture analyzed on SDS-PAGE (**Fig 1A**). Approximately 200 μg protein was obtained from 100 pairs of salivary glands, corresponding to 2 μg in a salivary gland pair.

Previous proteomics studies have identified over 120 individual proteins in female *Ae*. *aegypti* mosquito salivary gland with some proteins more abundant than others [10,35]. Our SDS-PAGE analysis of the whole SGE revealed the presence of at least 9 major intense protein bands and several minor protein bands (**Fig 1A).**

### Pull-down and identification of receptor-interacting SGPs

All receptor bait proteins were tagged with an Fc-tag except TLR4, which had 6×-histidine tag and was analyzed on SDS-PAGE (**Fig 1B**). The SGEs were precleared using regenerated protein A beads or nickel beads. The SGE flow-through was collected for subsequent pull-down experiments. Receptor bait proteins were bound to regenerated protein A beads, or nickel beads for TLR4 and then incubated with precleared SGE. The precleared SGE was first incubated with receptor bait proteins in a second experimental set-up, followed by incubation with the appropriate regenerated beads. After incubation, the beads from both set-ups were washed to remove unbound SGPs. Samples from neat precleared SGE (**Fig 1A**), bead-binding proteins excluded after preclearing, flow-through after incubation with receptor, and washes were boiled in SDS-dye and then analyzed on SDS-PAGE. Both the procedures yielded comparable results. The protein bands on SDS-PAGE were excised and analyzed using a bottom-up proteomic approach to identify receptor-binding SGPs. Many SGPs interacted with CD4, CD14, CD86, DC-SIGN, and TLR4 receptors (**Fig 1 and Table 1)**.

**Table 1:** Human receptor protein binding SGPs identified by pull-down and MS analysis.

Human receptor proteins	Bound SGPs	SGP ID	Mature MW (kDa)	Total number of peptides (Green-Red-Yellow)	Percent coverage
**CD4**	Long-form D7 protein 1	AAEL006417	36.2	9(9-0-0)	39.2
	AaVA-1	AAEL000793	26.7	7(6-1-0)	40.3
	NeST1	AAEL003601	36.2	4(3-1-0)	14.6
	Aegyptin	AAEL010235	27.0	2(2-0-0)	21.3
**CD14**	Apyrase	AAEL006347	60.2	9(8-1-0)	16.0
	Long-form D7 protein 1	AAEL006417	36.2	9(8-0-1)	36.1
	Aegyptin	AAEL010235	27.0	2(2-0-0)	17.6
**CD86**	Apyrase	AAEL006347	60.2	7(6-0-1)	12.3
	NeST1	AAEL003601	36.2	3(3-0-0)	10.4
	Long-form D7 protein 2	AAEL006424	35.1	11(9-0-2)	39.2
	Aegyptin	AAEL010235	27.0	2(2-0-0)	21.3
	Ficolin-3	AAEL000749	33.4	4(3-1-0)	11.4
**DC-SIGN**	Apyrase	AAEL006347	60.2	8(7-1-0)	13.0
	Long-form D7 protein 2	AAEL006424	35.1	5(5-0-0)	20.9
	AaVA-1	AAEL000793	26.7	6(5-0-1)	40.8
	SGS1	AAEL009993	380.0	1(1-0-0)	3.4
**TLR4**	Apyrase	AAEL006347	60.2	5(4-0-1)	7.7
	ADA2	AAEL005676	55.9	8(8-0-0)	25.6
	GAPDH	AAEL001593	39.3	1(1-0-0)	4.5
	Ficolin-3	AAEL000749	33.4	4(3-1-0)	11.4
	Malate dehydrogenase	AAEL008166	44.3	8(7-1-0)	32.6

SGPs: Salivary gland proteins; AaAVA-1: *Ae. aegypti* allergen 1; NeST1: Neutrophil stimulating protein 1; SGS1: *Aedes aegypti* salivary gland surface protein 1. GAPDH: glycerol-3-phosphate dehydrogenase. The green-red-yellow peptides are found in **S5–S10 Figs**. Green represents positive reliable peptides, yellow represents low confidence peptides and red represents very poor intensity peptides.

Four proteins, namely D7 (AAEL006417), *Ae*. *aegypti* venom allergen 1 (AaVA-1), neutrophil stimulating protein 1 (NeST1), and aegyptin were identified as bound to CD4 in pull-down experiments (**Fig 1C**). The peptides obtained for these proteins from the peptide mass fingerprint were 9, 7, 4, and 2, respectively (Table 1). However, some of these peptides are of low detection confidence for D7 (AAEL006417) and NeST1 (**S5 Fig and Table 1**).

Recombinant CD14 was associated with SGPs apyrase, D7 (AAEL006424), and aegyptin (**Fig 1D**) with respective total number of peptides being 9, 9, and 2 while some of these peptides were of low detection confidence for apyrase and D7 (AAEL006424) (**S6 Fig and Table1**).

Five SGPs were found associated with CD86, including some of the previously identified proteins such as apyrase, NeST1, D7 (AAEL006424), aegyptin and one unique protein, ficolin-3 (**Fig 1E**). The total peptide coverage for these proteins were 7, 3, 11, and 4, respectively. Some of the peptides were of low detection confidence for apyrase and D7 (AAEL006424) (**S7 Fig and Table 1**).

**Fig 2:**
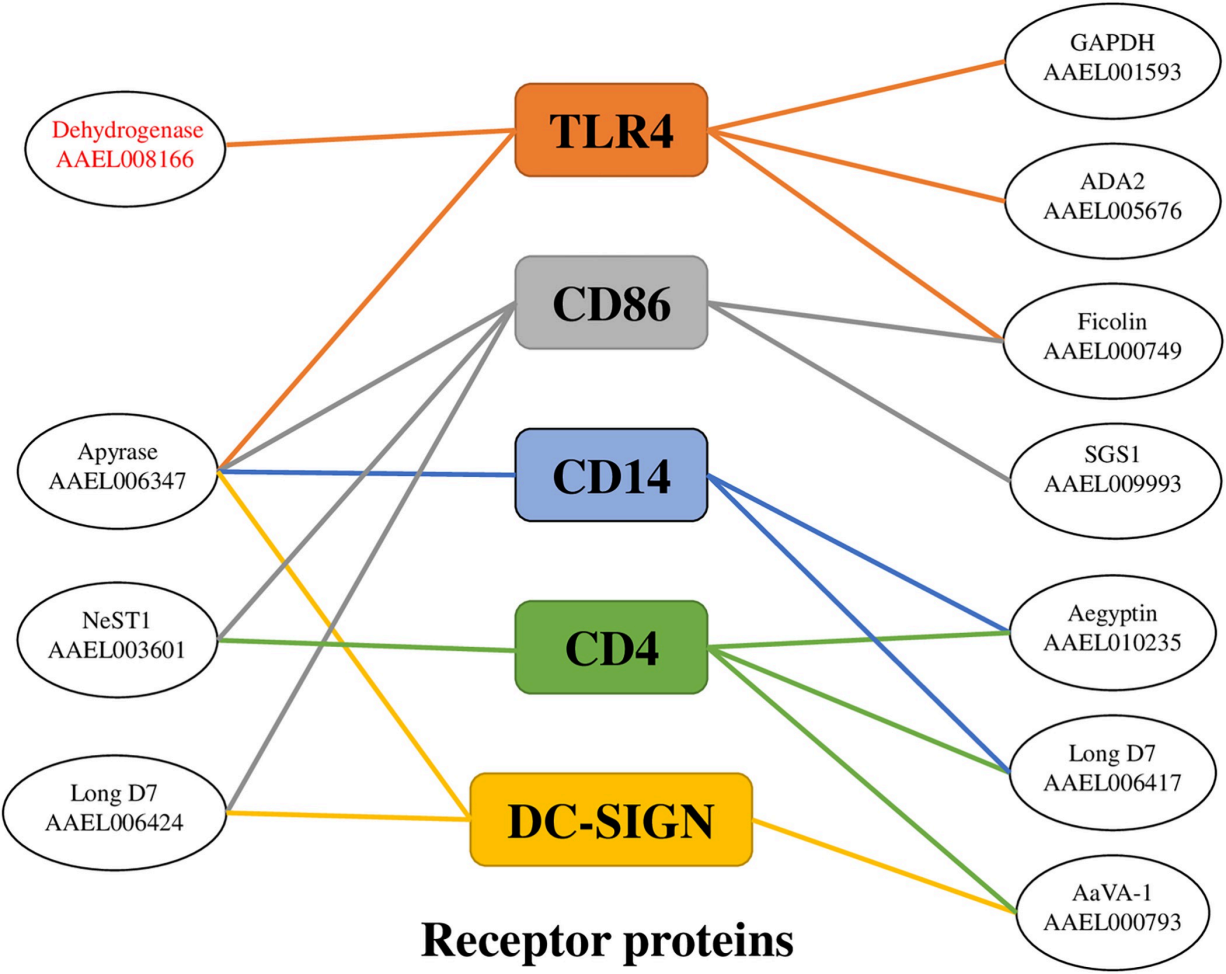
Human receptor proteins-SGPs interacting network. CD4, CD14, CD86, DC-SIGN and TLR4 are the human receptor proteins. All the SGPs identified here were previously reported to be present in the mosquito saliva [35]. Malate dehydrogenase (AAEL001593) has not been reported in the mosquito saliva.

Most of the previously identified SGPs such as apyrase, D7 (AAEL006424), and AaVA-1 were also identified with DC-SIGN as well as a unique protein known as the salivary gland surface protein 1 (SGS1) (**Fig 2F**). These proteins were detected by the mass spectrometry with a respective total number of peptide coverages being 8, 5, 6, and 1. However, some of the peptides were of low detection confidence for apyrase and AaVA-1 (**S8 and S9 Figs and Table 1**).

Similar to other receptor proteins, TLR4 also shows association with apyrase and ficolin-3, although it also bound to two unique proteins: adenosine deaminase 2 (ADA2) and glycerol-3-phosphate dehydrogenase (GAPDH) (**Fig 1G**) with total peptide coverages of 8 and 1, respectively (**S4 Fig and Table 1**). While malate dehydrogenase was also associated with TLR4 with total peptide coverage of 8, this protein has not been reported in the mosquito saliva (**S10 Fig** and **Table 1**).

Overall, we identified 11 SGPs **(Table 1)** that interacted with one or more of the human receptor proteins with a relatively high confidence level of the detected peptides, although a few peptides were of low confidence. We generated an interaction network to indicate the interaction of SGPs with one or more receptor proteins and the interaction of receptor proteins with different SGPs (**Fig 2**).

Some of the SGPs identified in the current study were previously shown to play essential roles in virus transmission or infection (**Table 2**), including ADA2, D7 proteins (AAEL006424 and AAEL006417), apyrase, AaVA-1, and NeST1. The D7 (AAEL006417), AaVA-1, and NeST1 were selected for further interaction validation with CD4 and DC-SIGN receptors.

**Table 2:** Roles of the receptor binding SGPs in virus transmission/infection.

SGE Factor	Role in infection	Mechanism	Ref.
Apyrase (AAEL006347)	May enhance viral infection	Inhibits ADP-dependent platelet aggregation	[36]
Long form D7 protein (AAEL006417)	Enhances dengue viral infection	Interacts with dengue virus. Binds biogenic amines and leukotrienes. Inhibits platelet aggregation	[3]
Long form D7 protein (AAEL006424)	Inhibits dengue replication	Binds to the envelope protein and virion	[35]
Aegypti venom allergen 1 (AaVA-1) (AAEL000793)	Enhances DENV and ZIKV viral replication in DCs and macrophages	Binds to autophagy inhibitor, LRPPRC. Promotes autophagy	[3]
Aegyptin (AAEL010235)	Modulates DENV infection. Inhibits clot formation during blood meal.	Elevates induction of immune response. Increases the concentrations of GM-CSF, IFN-γ, IL-5, and IL-6	[37]
Ficolin (AAEL000749)	Immunity-related	Detects carbohydrate molecules on pathogens such as viruses	[38]
Adenosine deaminase2 (AAEL005676)	Enhances viral replication	Inhibits IFN mRNA expression	[3]
GAPDH (AAEL001593)	May contribute to the appearance of steatosis in dengue-infected patients	Directly interact with DENV full-length non-structural protein 3 (NS3)	[39]
NeSt1 (AAEL003601)	Enhances ZIKV replication.	Induces expression of chemokines (pro-IL-1B, CXCL2, and CCL2)	[4]
SGS1 (AAEL009993)	Facilitates salivary gland invasion by ZIKV and *Plasmodium gallinaceum*	Unknown	[40,41]
Malate dehydrogenase (AEEL008166)	Unknown	Unknown	
AgBR1 (AEEL001965)	Enhances ZIKV infection	Suppresses inflammatory responses	[5]
LTRIN (AEEL017253)	Enhances ZIKV infection	Interacts with and prevents human LTßR dimerization	[8]

SGPs: Salivary gland proteins; AaVA-1: *Ae*. *aegypti* venom allergen 1; NeST1: Neutrophil stimulating protein 1; SGS1: *Aedes aegypti* salivary gland surface protein 1. GAPDH: glycerol-3-phosphate dehydrogenase.

### ELISA confirms AaVA-1, D7, NeST1, and AgBR1 directly interact with recombinant CD4 or DC-SIGN proteins

**Fig 3:**
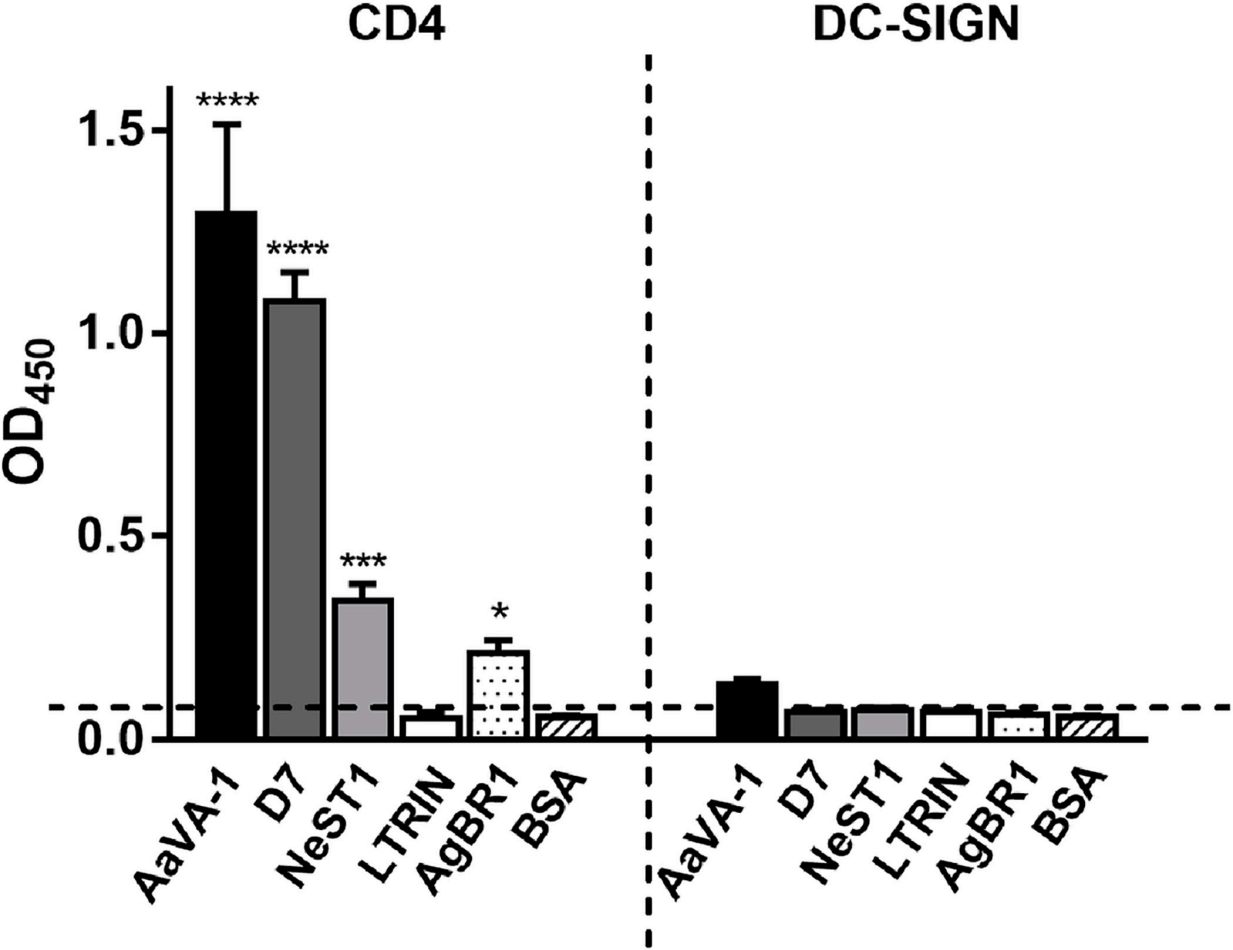
Interaction of recombinant ectodomain CD4 and DC-SIGN proteins with individual recombinant SGPs. ELISA results of SGPs interaction with CD4 protein and DC-SIGN protein. The mosquito SGPs include: AaVA-1, D7 (AAEL006417), Neutrophil stimulating protein 1 (NeST1), Lymphotoxin-β receptor inhibitor (LTRIN), and *Ae*. *aegypti* bacteria-responsive protein 1 (AgBR1). Bovine serum albumin (BSA) was used as a negative control. LTRIN and AgBR1 were not previously identified in our pull-down experiment. Statistical significance was calculated using the two-way ANOVA on GraphPad Prism version 7.0 and represented as p values: *p < 0.05, ***p < 0.001, ****p< 0.0001. The cut-off value of negative control was calculated as the mean + three times the standard deviation at the value of 0.066 which is shown the dashed line before applying the statistical analysis.

From the pull-down assays, AaVA-1, D7 (AAEL006417), and NeST1 were shown to associate with CD4 protein, with AaVA-1, also shown to associate with DC-SIGN (**Fig 1C and 1F**). To test these one-to-one interactions, we produced the extracellular domains of both CD4 and DC-SIGN and AaVA-1, D7 (AAEL006417), and NeST1 SGPs using the insect baculovirus expression system (**S1 Fig**). An ELISA assay was conducted with these receptor proteins. We confirmed that AaVA-1 and D7 (AEEL006417) showed significant interactions with the ectodomain of the recombinant CD4, whereas a moderate interaction was observed for NeST1 (**Fig 3**). AaVA-1which was pulled-down by DC-SIGN, poorly interacted with recombinant DC-SIGN (**Fig 3**). D7 (AEEL006417) and NeST1, not identified to interact with DC-SIGN in the initial pull-down, did not interact with DC-SIGN in the ELISA, suggesting the authenticity of the pull-down assays (**Fig 3**).

As mentioned earlier, tick saliva proteins such as Salp15 bind to the extracellular domain of human CD4 protein [15,16] via the last C-terminal 20-amino acids residues (P11 peptide). We conducted a sequence alignment for AaVA-1 and D7 (AEEL006417) against Salp15 (**S2 and S3 Figs**). AaVA-1 and D7(AEEL006417) exhibit only ~25% identity with Salp15. However, the key cysteine residues are conserved in these proteins, suggesting a similar fold; albeit the mode of interaction for AaVA-1 and D7 (AEEL006417) with CD4 is unknown. It will, therefore, be interesting to explore the roles of AaVA-1, D7 (AAEL006417), and NeST1 binding with CD4 in viral infection.

Other major *Ae*. *aegypti* mosquito saliva proteins, lymphotoxin-β receptor inhibitor (LTRIN), and *Ae*. *aegypti* bacteria-responsive protein 1 (AgBR1), which promote ZIKV infection both *in vitro* and *in vivo* [5,8] (**Table 2**) were not identified in our pull-down assay. However, these two proteins were also produced from the insect baculovirus expression systems (**S1 Fig**) and tested for interaction with CD4 and DC-SIGN in our ELISA assay. Whereas AgBR1 moderately interacts with CD4, LTRIN did not interact with CD4, and neither AgBR1 nor LTRIN interacted with DC-SIGN (**Fig 3**). The binding of these SGPs to human proteins may play pro-viral or anti-viral roles.

### Effects of AaVA-1, NeST1, or D7 (AEEL006417) proteins on CD4^+^ T cells

**Fig 4:**
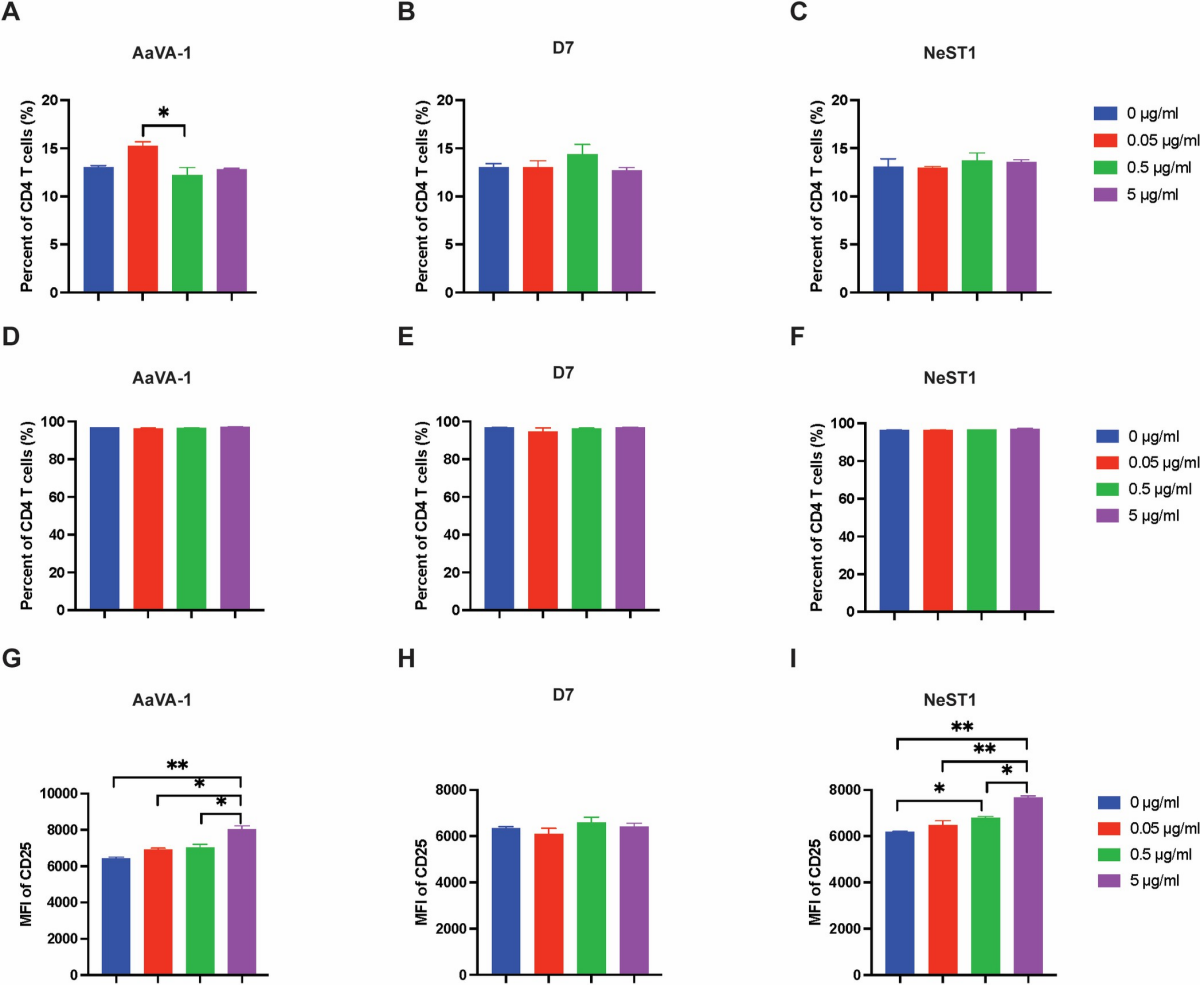
AaVA-1 and Nest1 enhance the activation of T cells. Human peripheral blood mononuclear cells (PBMC) with mosquito proteins at different concentration, 24 hours later activation marker CD69 and CD25 were measured. (**A-C**). Frequency of CD69 expression on CD4^+^ T cells was compared after different concentration of AaVA-1 (**A**), D7 **(AAEL006417)** (**B**), or Nest1 (**C**) treated PBMC cells. (**D-F**). Frequency of CD25 expression on CD4^+^ T cells was compared after different concentration of AVAa-1 (**D**), D7 **(AAEL006417)** (**E**), or Nest1 (**F**) treated PBMC cells. (**G-I**). Mean fluorescence of intensity of CD25 expression on CD4^+^ T cells was compared after different concentration of AaVA-1 (**G**), D7 **(AAEL006417)** (**H**), or NeST1 (**I**) treated PBMC cells.

Human peripheral blood mononuclear cells (PBMC) were incubated with mosquito proteins such as AaVA-1, NeST1, or D7 at different concentrations (0.05 μg/ml, 0.5 μg/ml, 5 μg/ml). 24 hours later activation marker CD69 and CD25 were measured by flow cytometry. The data showed that the frequency of activation markers expression on CD4^+^ T cells was not affected including CD69 and CD25 (**Fig 4A–4F**) by the treatment with mosquito proteins. Because almost all of the CD4^+^ are CD25-positive (**Fig 4A–4F**), we compared the mean fluorescent intensity of CD25 expression on CD4^+^ T cells. We found that AaVA-1 and NeST1 promoted CD25 expression on CD4^+^ T cells (**Fig 4G–4I**). These observations indicate that our proteins, including AaVA-1 and NeST1, can promote activation of CD4^+^ T cells.

## Discussion

Mosquito-borne pathogenic infections are among the leading causes of death globally. Part of the transmission and infection mechanism involves the interaction between mosquito saliva proteins with human proteins. Here, we conducted a pull-down assay and identified 11 SGPs from *Ae*. *aegypti* that interact with one or more human receptor proteins. We also confirmed some of these interactions with ELISA assay. The salivary gland of *Ae*. *aegypti* contains over 120 individual proteins of which some of these proteins, such as actin, serpin, apyrases, and D7 proteins, are more abundant than other proteins [35]. The 56/65 kDa and 31 kDa intense protein bands have been shown to contain seven and thirteen different proteins, dominated by apyrase and D7 family of proteins and serpins, respectively [42].

To determine if the SGPs we detected from our pull-down assay were not only correlated with their abundance, we also cross-verified our data with the LC-MS/MS analysis data on *Ae*. *aegypti* SGE as detailed by Conway and colleagues [35]. Notably, we did not identify abundant SGPs such as actin and serpin in our pull-down experiments, although apyrase and D7 proteins (AEEL006417 and AEEL006424) were identified. All the SGPs naturally exist with signal peptides (except SGS1), suggesting they exist as preproteins in the salivary gland tissues. Most of the proteins have molecular weights in the range 25–40 kDa, while a few falls within 50–70 kDa with SGS1 being the largest of 380 kDa (**Table 1**). The various identified SGPs, their role in infection/blood-feeding, and the possible impact of their interactions with receptor protein on viral infections are discussed below:

The two-long form D7 proteins (AAEL006424 and AAEL006417) were associated with distinct sets of receptors. D7(AAEL006424) interacts with DC-SIGN and CD86 while D7 (AAEL006417) interacts with CD4 and CD14. There is crystal structure for the D7 (AAEL006424: [PDB: 3DYE]) [43]. These two D7 proteins share 39% sequence identity with all the ten disulfide bonds completely conserved for both proteins (**S4 Fig**), suggesting a similar fold and mode of interaction with receptors. In a virus overlay protein binding assay, apyrase, the two D7 proteins (AAEL006424 and AAEL006417) and aegyptin were shown to bind to DENV [44]. The D7 (AAEL006424) inhibits DENV *in vitro* and *in vivo* by directly interacting with the virus envelope protein [35]. Interestingly, contrary to the D7 (AAEL006424), the upregulation of the D7 (AAEL006417), which also interacts with DENV correlates with enhanced DENV and ZIKV infection [3] (**Table 2**). While our ELISA assay shows that D7 (AAEL006417) directly interacts with recombinant CD4, the recombinant D7 protein did not affect activation markers, CD25 and CD69, on T cells. This suggests that the possible D7 protein-CD4 interaction-mediated impact on viral infections may be directly affected by CD4 protein rather than downstream immune molecule activation.

Apyrase interacts with four receptors (CD86, DC-SIGN, TLR4, and CD14), and aegyptin was associated with CD4, CD14 and CD86. Both apyrase and D7 proteins (AAEL006424 and AAEL006417) bind to ADP which prevents platelet aggregation and blood clotting [36]. Similarly, aegyptin binds to collagen, inhibits platelet aggregation, and facilitate blood feeding [37]. Aegyptin was shown to be downregulated during DENV infection and reduces DENV titers at the bite site [37]. ADA2 exclusively interacts with TLR4. ADA2 also removes adenosine from bite site and aids in a prolonged blood-feeding [45] and has been shown to partially enhance dengue and Zika virus infection in human THP-1 cells [3]. Recently, apyrase was shown to directly interact with ZIKV [46]. The above analysis suggests the importance of **1)** the interactions of vector SGPs such as ADA2, D7 proteins (AAEL006424 and AAEL006417), apyrase, and aegyptin, with host receptors and **2)** the interaction of some SGPs with the pathogen/host molecules. These vector-host-pathogen interactions might impact blood-feeding and infection.

AaVA-1 was identified as interacting with CD4 and DC-SIGN in our pull-down experiments. More importantly, the direct and strong interaction of AaVA-1 with CD4 could be an interesting mechanism to explore in terms of impact on CD4^+^ T cells expression and viral infections, a mechanism employed by the tick protein Salp15 in suppressing human immune system and facilitating the infection of the Lyme disease-causing agent *Borrelia burgdorferi* bacterial infection [47]. AaVA-1 is a crucial target that promotes DENV and ZIKV infection in vitro in human THP-1 cells and in vivo in mouse models via interaction with human leucine-rich pentatricopeptide repeat-containing protein (LRPPRC) [3]. Recently, AaVA-1 was shown to directly interact with ZIKV [46]. This suggests the potential role of AaVA-1-CD4 and AaVA-1-DC-SIGN interactions on viral infection in human hosts. Surprisingly, however, similar to the D7 protein, recombinant AaVA-1 protein did not affect activation markers, CD25 and CD69 on T cells. This suggests that the possible AaVA-1-CD4 interaction-mediated impact on viral infections may be associated with the direct effect on CD4 protein rather than downstream immune molecule activations. Moreover, the ability of AaVA-1 and NeST1 proteins to enhance the activation of CD4^+^ T cells suggest that these proteins might play a role in the immune response involved in mosquito-borne viral infections.

NeST1, GAPDH, and ficolin-3 were identified with TLR4, while NeST1 and ficolin-3 were also bound to CD86 receptor in our pull-down experiments. NeST1 further interacts with CD4. NeSt1 activates primary mouse neutrophils and enhances ZIKV infection [4]. Ficolin-3 is a humoral molecule of the innate immune systems which detects carbohydrate molecules on pathogens such as viruses [48]. A recent study shows that *Plasmodium* invasion induces ficolin expression [49].

Notably, human TLR4 interacts with DENV non-structural protein 1 (NS1) on endothelial cells and contributes to vascular leakage in dengue shock [50]. TLR4 also directly interacts with DENV NS3, resulting in a reduced glycolytic activity of GAPDH [39,51]. This suggests a potential impact of NeST1-, GAPDH-and ficolin-3-TLR4 interactions on viral infections.

SGS1was associated with only CD86. Although the molecular weight of the protein band containing the SGS1 was lower (50–55 kDa) than the theoretical full-length protein (380 kDa), the MS data suggests that this might contain a cleaved N-terminal and C-terminal region of the SGS1 protein. SGS proteins are major immunogenic proteins implicated in *Plasmodium* parasites infection of mosquito salivary gland tissues [40]. Other SGS families such as SGS3, SGS4 and SGS6 are malaria blocking targets in *Anopheles* mosquitoes [52].

**Fig 5:**
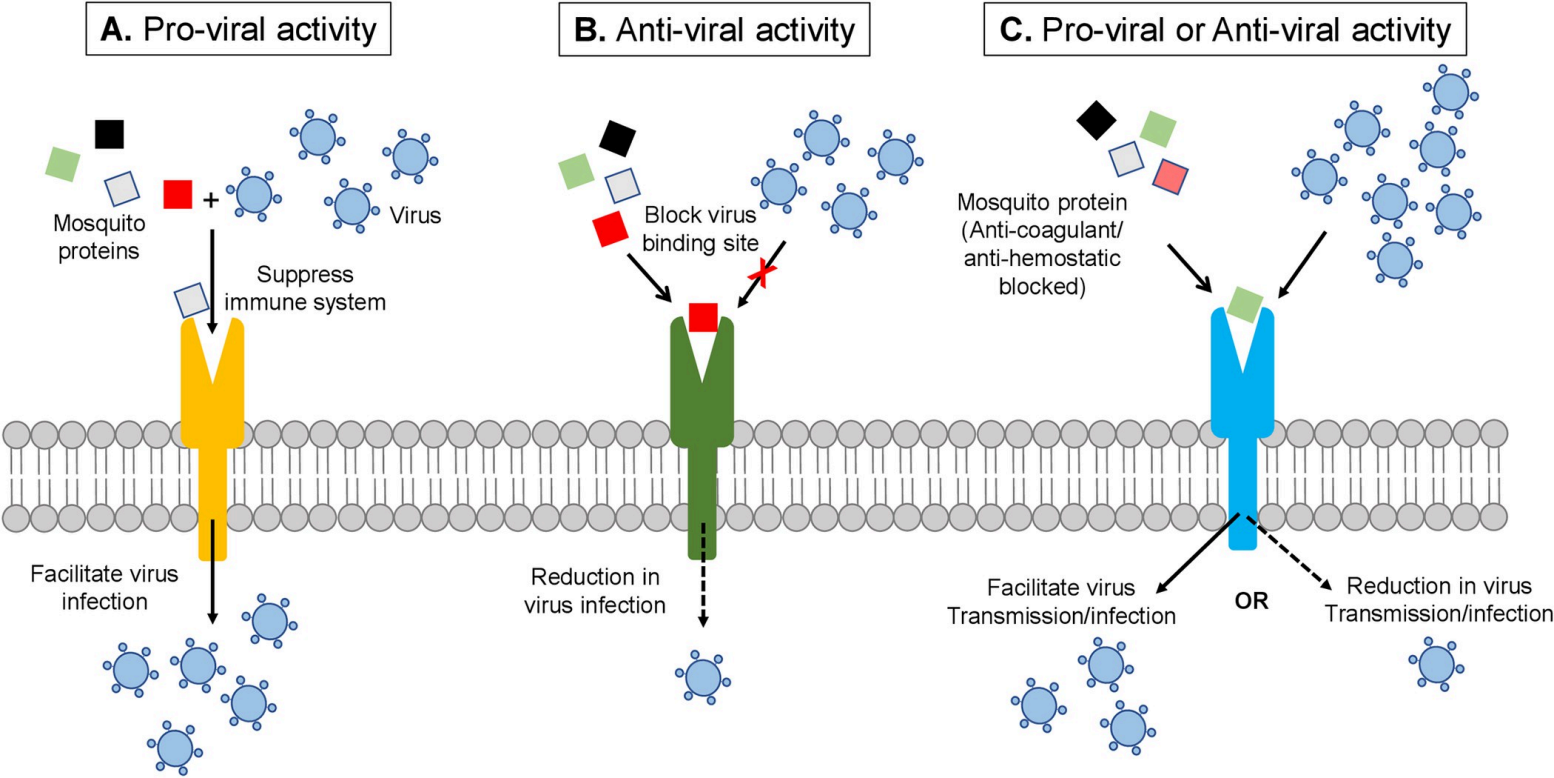
Schematics of the potential role of SGPs interactions with human host receptor proteins on pathogen transmission and or infection. (**A**) SGP binds to human immune receptor proteins resulting in host immunosuppression, thereby, promoting viral infection and replication. (**B**) SGP binds to human receptor protein and blocks the binding site of invading pathogens on the receptor resulting in the abrogation of pathogen cellular entry and reduced overall infection. (**C**) Anticoagulant SGP or proteins with anti-hemostatic activities hijacked by human receptor making it possible for blood to clot easily at the bite site resulting in the difficult acquisition of blood by feeding mosquitoes. This may cause the mosquito to inject more pathogen-infected saliva which could lead to enhanced pathogen transmission. Conversely, excessive blood clot could deter mosquito from further feeding resulting in a lower deposition of pathogens and lower transmission or infection.

The expression of the above-discussed proteins is regulated depending on specific viral infections. For example, apyrase, AaVA-1, and aegyptin were downregulated in response to DENV, ZIKV, and CHIKV infections. D7 proteins (AAEL006424 and AAEL006417) were upregulated in DENV infections but downregulated in ZIKV and CHIKV infections. SGS1 is downregulated in ZIKV and CHIKV infections, while ADA2 is upregulated in ZIKV and CHIKV infections [53]. As such, the interactions of SGPs with human proteins can potentially impact the infection process of these viruses in human hosts by playing pro-viral or anti-viral roles (**Fig 5**).

### Conclusion

Our study identified 11 mosquito SGPs that interact with one or more human receptor proteins. Different protein families were identified, including odorant-binding D7 family of proteins, apyrase, SGS1, NeST1, AaVA-1, and dehydrogenase enzyme. Notably, all the receptor-interacting SGPs identified from this study were previously found in the saliva expectorate of female mosquitoes, (except the malate dehydrogenase and Glycerol-3-phosphate dehydrogenase), suggesting a potential downstream interaction with the above receptors. The impact of the direct interaction of salivary proteins and human receptor proteins on virus infection should be explored as this may either play pro-viral or anti-viral roles. Understanding the newly identified interacting partners might offer more insight into the complex molecular mechanism that hematophagous arthropod proteins employ in viral transmission. Our work provides a framework for the future exploration of mosquito proteins-human host proteins interactions and how this can be leveraged to curb mosquito-borne diseases.

### Limitations

While our studies seek to establish a foundation in vector-host interactions, we would like to identify the following limitations and caveats considering the challenges associated with working with mosquito saliva proteins:

1. A limitation of our pull-down assay is the preclearing stage which eliminates bead-binding proteins that might be important interacting partners for the human receptor proteins. However, this step was necessary to exclude the SGPs that might non-specifically bind to the agarose beads.
2. The pull-down assay alone is insufficient to differentiate SGPs that directly interact with human receptor proteins *versus* SGPs that indirectly interact with receptor proteins by binding to other SGPs, hence the need for further validation. Thus, false positives/negatives or non-specific binding may also be an inherent limitation of this experiment. As such, individual one-to-one interaction studies with each salivary protein will be necessary to validate the various putative bindings observed.
3. Moreover, because majority of the more than 120 mosquito saliva proteins identified so far are of less than or around 100 kDa, we optimized our SDS-PAGE such that the run attempts to capture smaller molecular weight proteins. To achieve this, we stopped the SDS-PAGE run earlier than expected. This introduces a limitation of not being able to identify a few higher (>100 kDa) molecular weight proteins.
4. To reduce the number of bands that had to be taken for mass spectrometry analysis (as some individual bands are too faint), some of the closely associated bands or bigger gel slabs were excised together and taken for the MS analysis.
5. The amount of recombinant salivary proteins used on human PBMC may not be representative of the amount injected by the mosquito during bite episodes. It is difficult to calculate the exact amount of individual protein injected at any point in time. This presents an inherent limitation in in vitro biochemical assays that attempts to mimic the effect of proteins injected by mosquito into the host. It is, therefore, inconclusive that the amount of respective native proteins present in saliva would induce similar phenotypes as observed in this study or similar biochemical studies.

## Materials and methods

### Mosquito rearing and salivary gland dissection

Over 2500 uninfected female *Ae*. *aegypti* mosquitoes were provided by the Duke-NUS medical school insectary, Singapore. Mosquitoes were maintained on a sugar solution at 27°C and 80% humidity according to standard rearing procedures. Salivary glands were isolated as described previously [35]. Briefly, mosquitoes were anesthetized on ice to immobilize them for dissection. With the aid of dissection kits and microscope, a pair of glands was isolated from each mosquito and washed in phosphate-buffered saline. 100 salivary glands were stored per tube in 100 μl sterile phosphate-buffered saline (PBS) supplemented with protease inhibitor tablet (Roche) and homogenization beads and this was kept at -80°C until use.

### Gland extract preparation

100 μl lysis buffer (1.5 mM MgCl_2_, 10 mM tris HCl, 10 mM NaCl, 1% Nonidet P-40) was added to salivary glands stored in PBS containing protease inhibitor tablet and homogenization beads. Glands were homogenized using a tissue homogenize (Biogear, Life Sciences laboratory). Sample was then spun down at 12,600 g for 15 min at 4°C and supernatant collected in clean tube as salivary gland extract (SGE). SGE protein was concentrated at 10,000 g and protein quantified using Bradford method. Salivary gland proteins were stored at-20°C until use.

### Preparation of protein A beads

100 μl of protein A beads in 50% suspension was transferred into a 2 ml microcentrifuge tube. Beads were then equilibrated by adding 5 volumes of PBS, mix, and centrifuge for 30 s at 2,000 g and the supernatant carefully removed using a micropipette. The step was repeated 3x.

### Preclearing salivary gland extract (SGE)

To preclear SGEs with protein A beads, SGE were added to new equilibrated beads and incubated at 4°C for 1 h with gentle end-to-end mixing. Pre-cleared lysate was then micro-centrifuged at 14000 g for 20 seconds to pellet Protein A. Supernatant was collected as precleared SGE. Proteins bound to beads are regarded as proteins non-specifically bound. The proteins in this control allow us to subtract proteins binding non-specifically to beads.

### Methods of forming SGE-receptor complexes

#### Complexing protein baits with protein A beads first

Five human receptor proteins (CD4, CD14, CD86 and DC-SIGN with Fc-tags) and TLR4 with 6XHis-tag were used as baits. Receptor bait proteins were purchased from Sinobiologicals and Afirmus Biosource (produced using the mammalian HEK expression system). 100 μg/300 μl of each bait protein was added to equilibrated beads at 4°C for 1 h. Samples were spun down at 4°C for 30s, 2000 g and supernatant collected and labelled in a new tube. Samples were then further washed 2x with 1 mL PBS.

Next, pre-cleared SGEs were added to beads-plus-protein-baits and incubated at 4°C for 1 h with end-to-end gentle rocking. Samples were then spun down gently at 2000 g for 30s and the flow through collected and labelled in a new tube. Unbound proteins were washed with 1 mL PBS. Samples were mixed and placed on magnetic stand for 30s. The supernatant was removed while tube was still on magnetic stand. The first wash was collected in a clean tube. Washes were repeated 2 more times. 100 μL of SDS-PAGE loading dye was then added to each bead-bait-SGP mixture and boiled at 70°C for 10mins. Boiled samples were allowed to cool for 5 mins, spun down and supernatant run on 12.5% SDS-PAGE gel. Well separated protein bands were cut and sent for protein identification using mass spectrometry.

#### Complexing SGE with receptor proteins first

Precleared SGE was incubated first with the receptor proteins and the complex incubated with the appropriate regenerated beads. The beads-receptor protein-SGE mixture was then thoroughly washed to remove unbound SGPs. The remaining bead mixture was then boiled and analysed on SDS-PAGE as previously described. The protein bands were further verified with mass spectrometry analysis to identify receptor-binding proteins.

### Recombinant protein expression and purification

The ectodomain of human CD4 (26–396 amino acid residues) and DC-SIGN (58–380 amino acid residues) and mosquito proteins, *Ae*. *aegypti* venom allergen 1(AaVA-1), D7 (AAEL006417), neutrophile stimulating protein 1 (NeST1), Lymphotoxin-β receptor inhibitor (LTRIN) and *Ae*. *aegypti* bacteria-responsive protein 1 (AgBR1) were cloned into the baculovirus expression vector pfastbac1 (Genscript) with GP64 signal peptide for protein secretion and 8His purification tag and a preScission protease cleavage site. Recombinant proteins were expressed in insect baculoviral expression system using *Spodoptera frugiperda* (SF9) cells. Secreted proteins were purified with nickel affinity chromatography and washed with buffer A (50 Mm Tris 8.0, 400 Mm NaCl, 5% glycerol, 20 Mm imidazole supplemented with protease inhibitor tablet (Roche). The protein was eluted with buffer B (50 Mm Tris 8.0, 300 Mm NaCl, 5% glycerol, 500 Mm imidazole supplemented with protease inhibitor tablet). The eluted protein was buffer exchanged into buffer C (50 Mm Tris 8.0, 400 Mm NaCl, 5% glycerol) and the 8His tag was cleaved off with preScission protease enzyme and the proteins were further purified to homogeneity using ion exchange and gel filtration chromatography.

### Enzyme-linked immunosorbent assay

High binding 96-well plates (Thermo Fisher Sci, MA) were coated overnight at 4°C with a 5 μg of all salivary gland proteins (AaVA-1, D7, NeST1, AgBR1, and LTRIN) constructs or 5 μg BSA in triplicated in coating buffer. The next day, plates were washed twice with PBS and blocked with blocking buffer (1% BSA in 1XPBST) for 1 h at 28°C. Plates were incubated with 1μg CD4 or DC-SIGN protein at 37°C for 1 hour, considering the temperature of the human body. Unbound CD4 or DC-SIGN receptor proteins were washed three times with buffer (1X PBST+ 0.01% Tween 20) and incubated with 100 μL of either 1 μg/mL rabbit anti-CD4 monoclonal antibody (Sinobiological, 10400-R104) or rabbit anti-DC-SIGN monoclonal antibody (HRP) (Sinobiologicals, 10200-R059-H), for 2 h at RT. Unbound antibodies were then washed three times and six times, respectively, with buffer (1X PBST+ 0.01% Tween 20) and the CD4 plates incubated with 100 μL of 1:5000 rabbit anti-mouse HRP conjugated secondary antibody and washed six times after 1 h incubation at RT. Reactions were visualized after incubating with 80 μL of tetramethylbenzidine (TMB). After 5 min the reaction was stopped with 1 M HCl, and plates were read at 450 nm in Tecan Infinite 200 pro (Life Sciences and Diagnostics).

### Effects of AaVA-1, NeST1, or D7 proteins on T cells activation

Human PBMCs from healthy donor’s whole blood or apheresis cone from Health Sciences Authority, Singapore, were obtained. Written informed consent was obtained from donors.

#### Flow cytometry

For cell-surface staining, cells were blocked with CD16/32 FcR-block (BioLegend, San Diego, CA) for 15 min, and stained with fluorescent dye-conjugated mAb for 30 min at 4°C. PE-anti-human-CD25 (M-A251), APC-anti-human-CD69 (FN50), were purchased from BioLegend (San Diego, CA).

#### Co-culture assay

PBMCs were isolated with Ficoll-Hypaque (GE Healthcare, Chicago, IL) density gradient centrifugation. Plasma was collected. Erythrocytes were removed with ACK solution for 5–7 mins. Cells were washed with RPMI 1640 twice and then seeded into anti-human CD3 (2.5 μg/ml) and anti-human CD28 (5 μg/ml) pre-coated plates in the presence of human IL-2 (100 IU/ml). For stimulation, various concentrations (0.05 μg/ml, 0.5 μg/ml, 5 μg/ml) of individual proteins were added to culture. 24 hours later, cells were harvested and cell surface marker expressions were measured by flow cytometry.

#### Statistical analysis

For the ELISA assay, statistical significance was calculated using the two-way ANOVA on GraphPad Prism version 7.0 and represented as p values: *p < 0.05, ***p < 0.001, ****p< 0.0001. For the cell line studies, multiple comparisons were performed using one-way ANOVA with Tukey’s post-hoc test when comparing among all groups. Data were shown as mean ± SEM, and *p* <0 .05 was considered statistically significant. * Indicates *p* < 0.05, ** indicates *p* < 0.01, *** indicates *p* < 0.001. The cut-off value of negative control was calculated as the mean + three times the standard deviation at the value of 0.066 which is shown the dashed line before applying the statistical analysis.

## Supporting information

S1 Fig Expression and purification of human receptor proteins CD4 and DC-SIGN and SGPs. (**A-B**) SDS-PAGE of the extracellular domains of human CD4 and DC-SIGN proteins. (**C-G**) SDS-PAGE of SGPs: AaVA-1, D7, NeST1, AgBR1 and LTRIN, respectively. All the proteins were produced from insect baculovirus protein expression system.
(TIF)

S2 Fig Sequence alignment between mosquito saliva protein AaVA-1 and tick saliva protein Salp15. Conserved amino acid residues white font and red background while similar residues have red font and white background. The conserved cysteine amino acid residues are indicated with black arrows. The boxed C-terminal region indicated the region of Salp15 that interacts with human CD4 protein.
(TIF)

S3 Fig Sequence alignment between mosquito saliva protein D7(AAEL006417) and tick saliva protein Salp15. Conserved amino acid residues white font and red background while similar residues have red font and white background. The conserved cysteine amino acid residues are indicated with black arrows. The boxed C-terminal region indicated the region of Salp15 that interacts with human CD4 protein.
(TIF)

S4 Fig Sequence alignment between mosquito saliva D7 proteins (*AAEL006417* and *AAEL006424* [PDB: 3DYE]). Conserved amino acid residues are shown with white font and red background while similar residues have red font and white background. The conserved disulphide bonds are indicated with black lines.
(TIF)

S5 Fig Percentage peptide coverage of SGPs from mass spectrometry output. The individual SGPs associated with CD4 human receptor protein and unique peptide coverages are provided. The green peptides represent positive reliable peptides of the proteins with high confidence making the overall percentage coverage indicated by each SGP while the red peptides represent very poor intensity and yellow represents low confidence intensity. The grey areas represent undetected regions forming majority of the peptides.
(TIF)

S6 Fig Percentage peptide coverage of SGPs from mass spectrometry output. The individual SGPs associated with CD14 human receptor protein and unique peptide coverages are provided. The green peptides represent positive reliable peptides of the proteins with high confidence making the overall percentage coverage indicated by each SGP while the red peptides represent very poor intensity and yellow represents low confidence intensity. The grey areas represent undetected regions forming majority of the peptides.
(TIF)

S7 Fig Percentage peptide coverage of SGPs from mass spectrometry output. The individual SGPs associated with CD86 human receptor protein and unique peptide coverages are provided. The green peptides represent positive reliable peptides of the proteins with high confidence making the overall percentage coverage indicated by each SGP while the red peptides represent very poor intensity and yellow represents low confidence intensity. The grey areas represent undetected regions forming majority of the peptides.
(TIF)

S8 Fig Percentage peptide coverage of SGPs from mass spectrometry output. The individual SGPs associated with DC-SIGN human receptor protein and unique peptide coverages are provided. The green peptides represent positive reliable peptides of the proteins with high confidence making the overall percentage coverage indicated by each SGP while the red peptides represent very poor intensity and yellow represents low confidence intensity. The grey areas represent undetected regions forming majority of the peptides.
(TIF)

S9 Fig Percentage peptide coverage of SGPs from mass spectrometry output. Sequence of the SGP associated with DC-SIGN human receptor protein and unique peptide coverages are provided. The green peptides represent positive reliable peptides of the proteins with high confidence making the overall percentage coverage indicated by each SGP while the red peptides represent very poor intensity and yellow represents low confidence intensity. The grey areas represent undetected regions forming majority of the peptides. Possible glycosylation/post-translational modification sites in SGS1 which could account for low peptide identification are indicated with boldened blue font (NXX), where XX represents two amino acid residues after the asparagine (N).
(TIF)

S10 Fig Percentage peptide coverage of SGPs from mass spectrometry output. The individual SGPs associated with TLR4 human receptor protein and unique peptide coverages are provided. The green peptides represent positive reliable peptides of the proteins with high confidence making the overall percentage coverage indicated by each SGP while the red peptides represent very poor intensity and yellow represents low confidence intensity. The grey areas represent undetected regions forming majority of the peptides.
(TIF)
