# Supplementary figures and images for: Identification of Aedes aegypti salivary gland proteins interacting with human immune receptor proteins
Source: PLoS Negl Trop Dis. 2022 Sep 7;16(9):e0010743. doi: 10.1371/journal.pntd.0010743 (PMC9484696; doi:10.1371/journal.pntd.0010743)

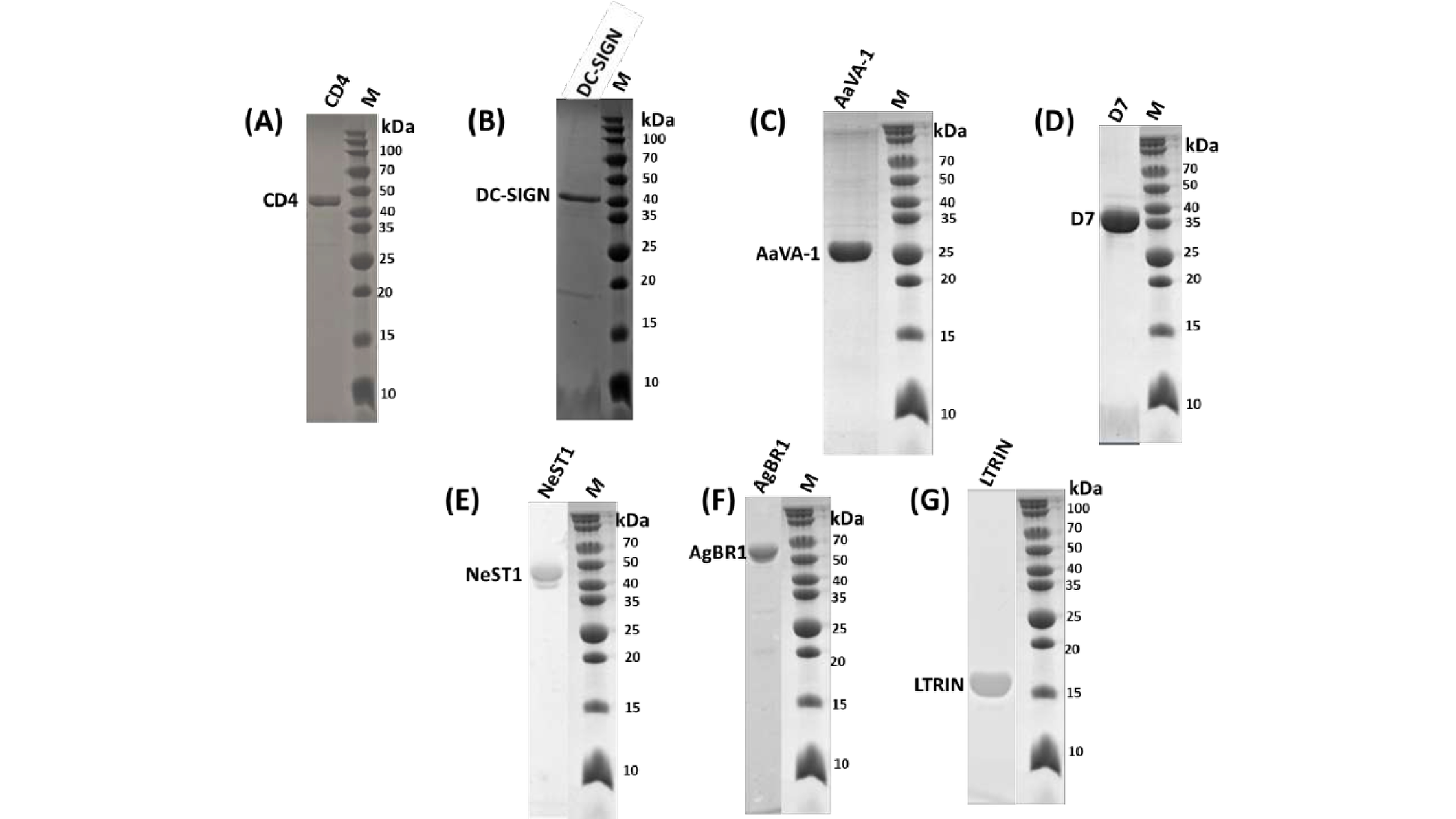

Supplement: S1 Fig — (A-B) SDS-PAGE of the extracellular domains of human CD4 and DC-SIGN proteins. (C-G) SDS-PAGE of SGPs: AaVA-1, D7, NeST1, AgBR1 and LTRIN, respectively. All the proteins were produced from insect baculovirus protein expression system. (TIF) [file pntd.0010743.s001.tif]

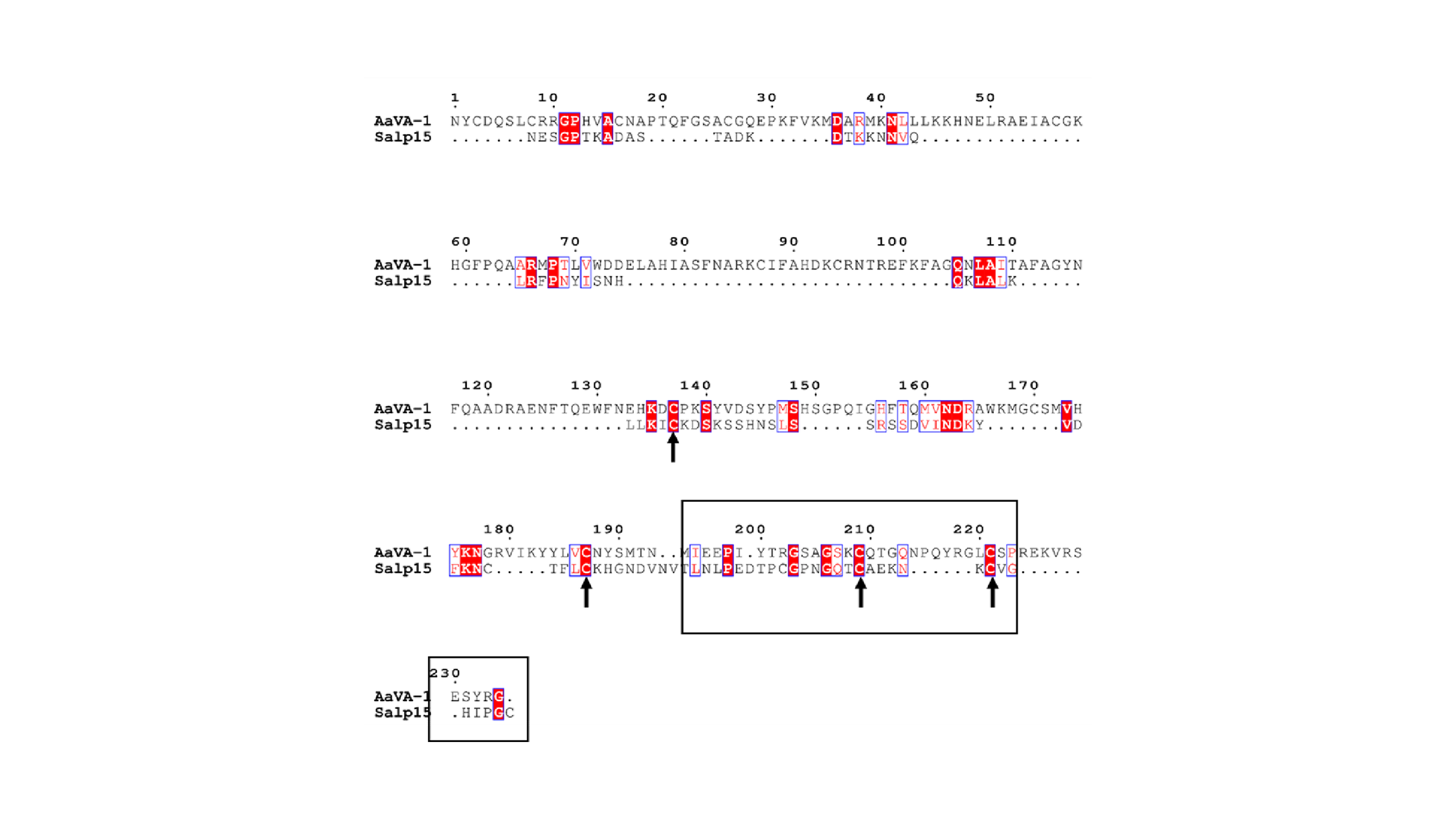

Supplement: S2 Fig — Conserved amino acid residues white font and red background while similar residues have red font and white background. The conserved cysteine amino acid residues are indicated with black arrows. The boxed C-terminal region indicated the region of Salp15 that interacts with human CD4 protein. (TIF) [file pntd.0010743.s002.tif]

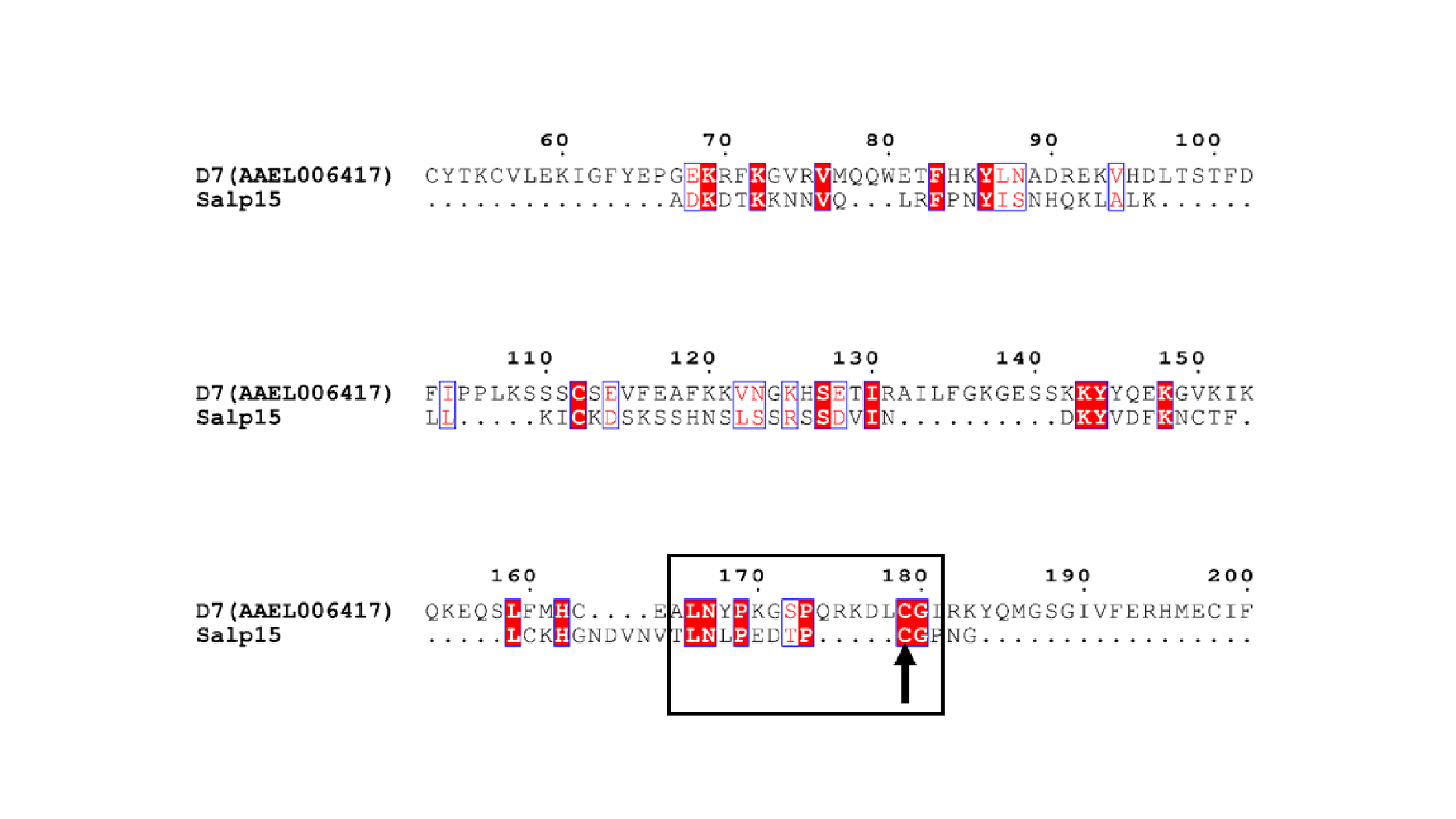

Supplement: S3 Fig — Conserved amino acid residues white font and red background while similar residues have red font and white background. The conserved cysteine amino acid residues are indicated with black arrows. The boxed C-terminal region indicated the region of Salp15 that interacts with human CD4 protein. (TIF) [file pntd.0010743.s003.tif]

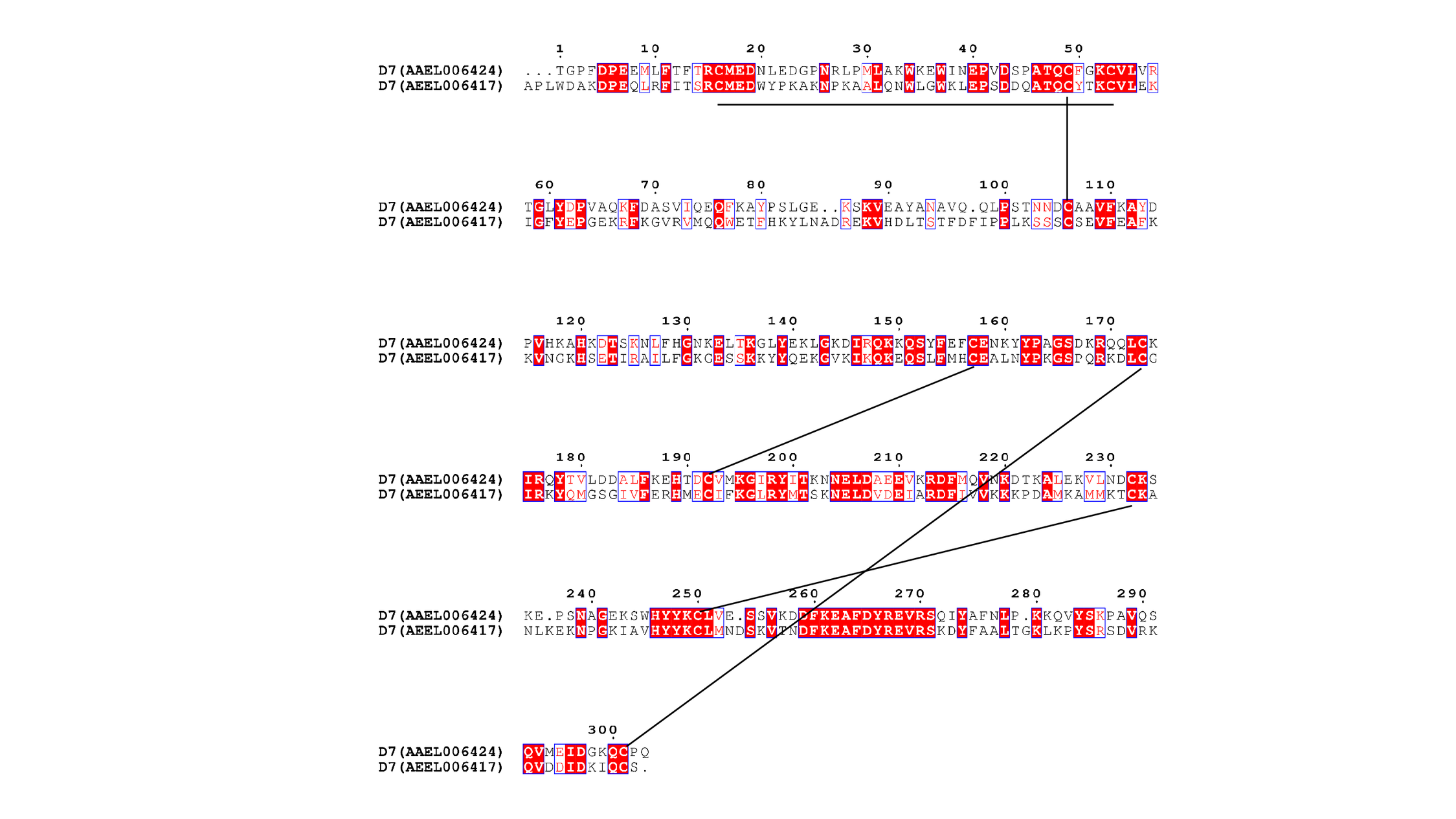

Supplement: S4 Fig — Conserved amino acid residues are shown with white font and red background while similar residues have red font and white background. The conserved disulphide bonds are indicated with black lines. (TIF) [file pntd.0010743.s004.tif]

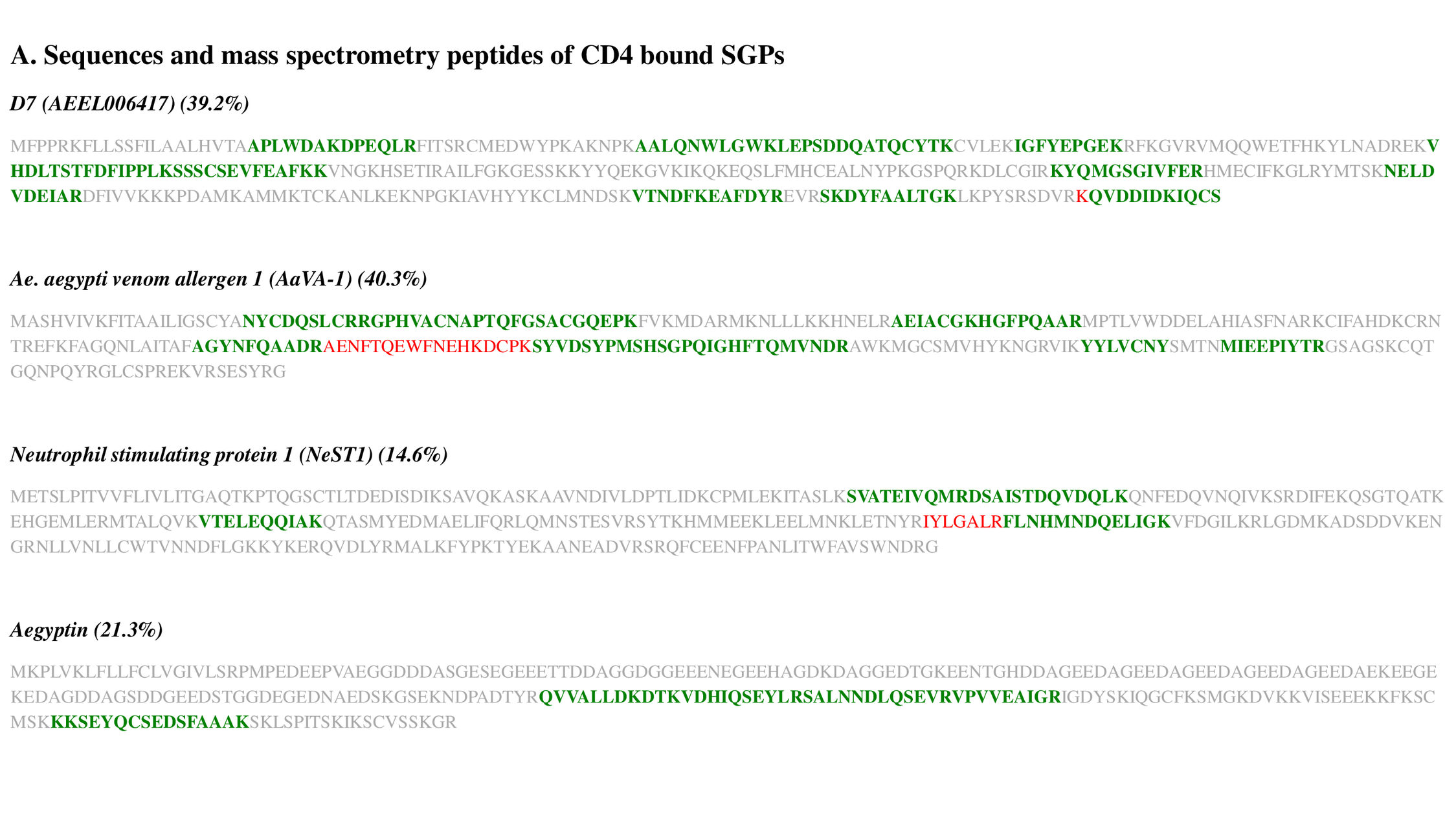

Supplement: S5 Fig — The individual SGPs associated with CD4 human receptor protein and unique peptide coverages are provided. The green peptides represent positive reliable peptides of the proteins with high confidence making the overall percentage coverage indicated by each SGP while the red peptides represent very poor intensity and yellow represents low confidence intensity. The grey areas represent undetected regions forming majority of the peptides. (TIF) [file pntd.0010743.s005.tif]

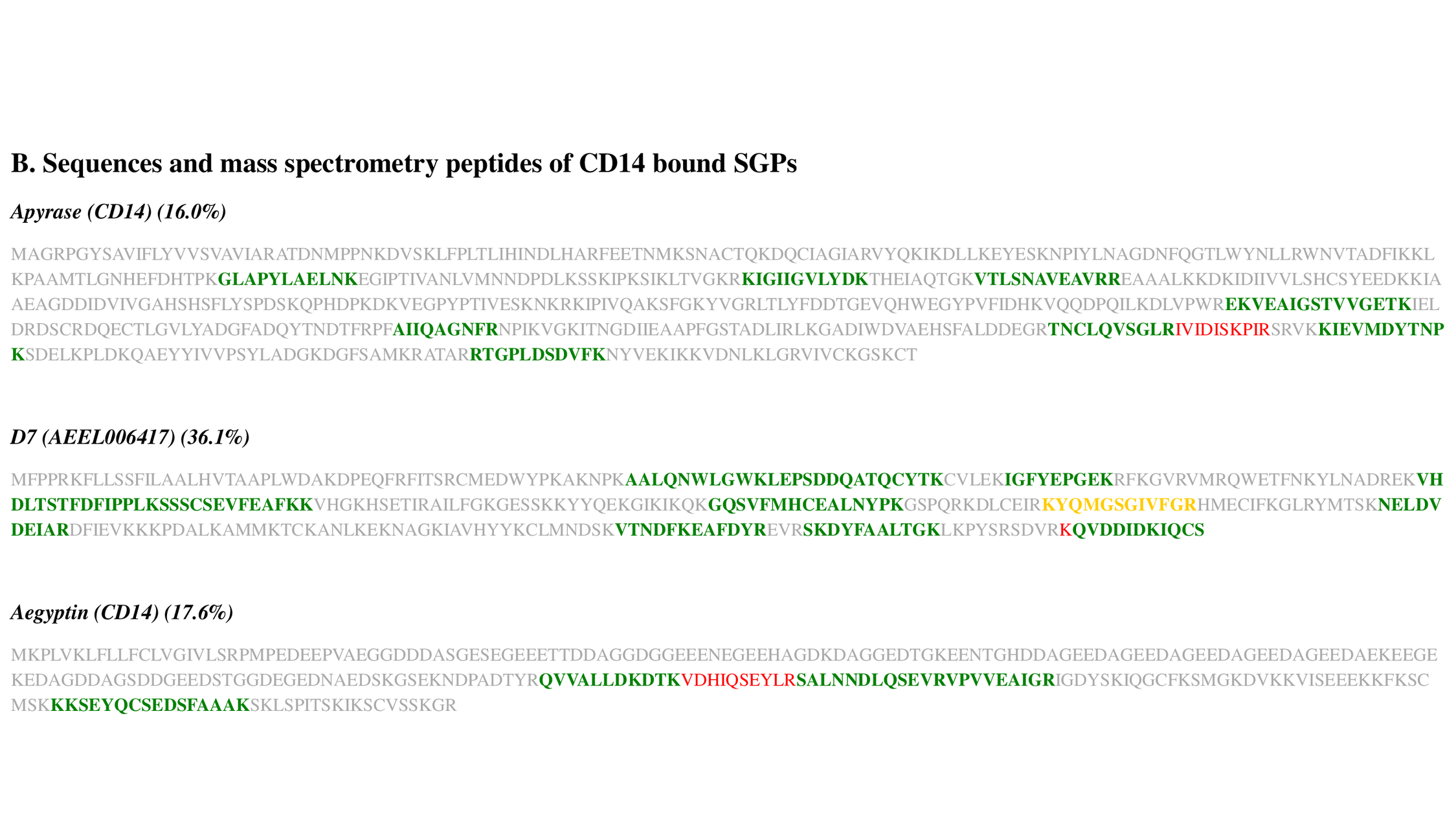

Supplement: S6 Fig — The individual SGPs associated with CD14 human receptor protein and unique peptide coverages are provided. The green peptides represent positive reliable peptides of the proteins with high confidence making the overall percentage coverage indicated by each SGP while the red peptides represent very poor intensity and yellow represents low confidence intensity. The grey areas represent undetected regions forming majority of the peptides. (TIF) [file pntd.0010743.s006.tif]

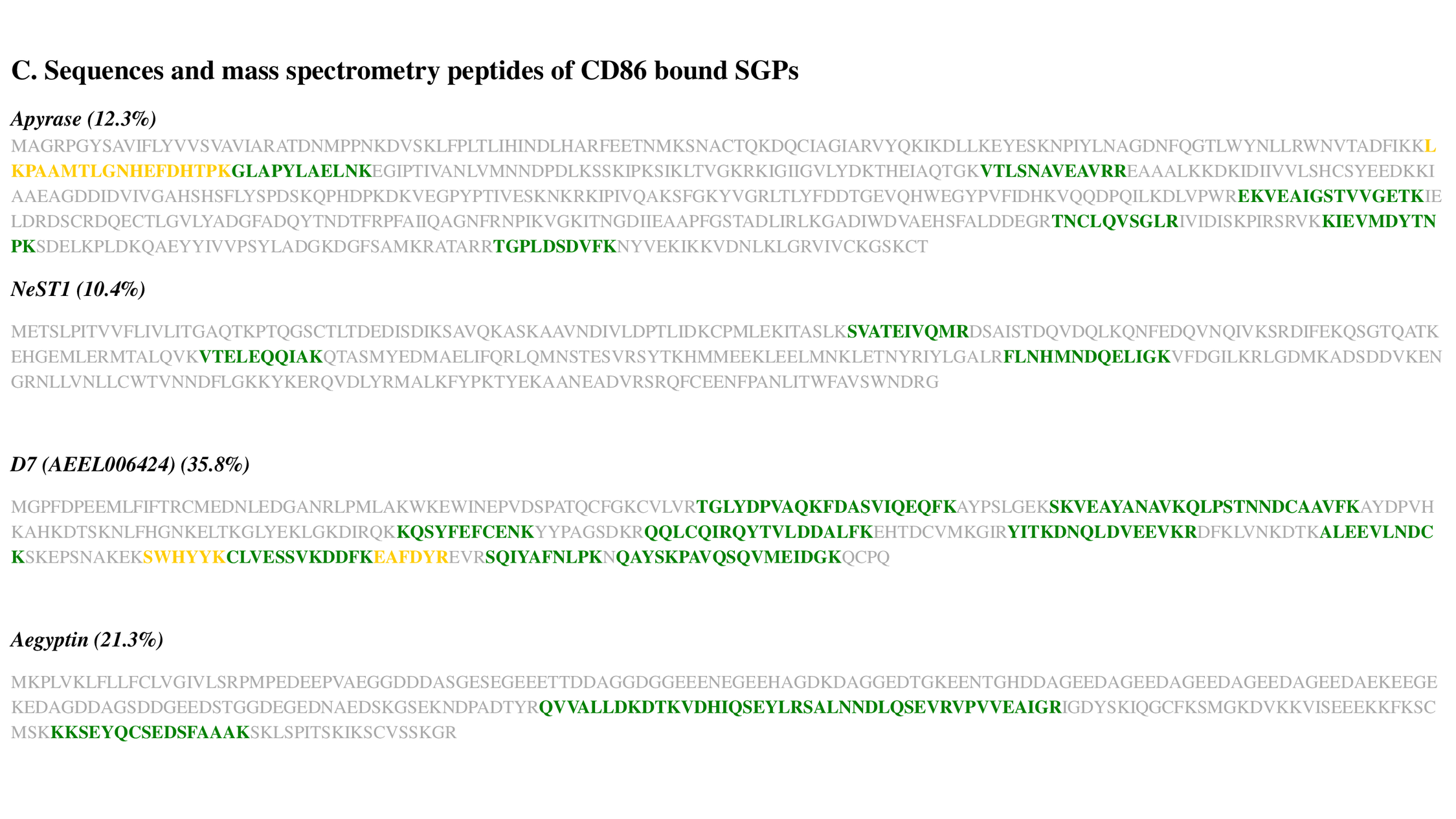

Supplement: S7 Fig — The individual SGPs associated with CD86 human receptor protein and unique peptide coverages are provided. The green peptides represent positive reliable peptides of the proteins with high confidence making the overall percentage coverage indicated by each SGP while the red peptides represent very poor intensity and yellow represents low confidence intensity. The grey areas represent undetected regions forming majority of the peptides. (TIF) [file pntd.0010743.s007.tif]

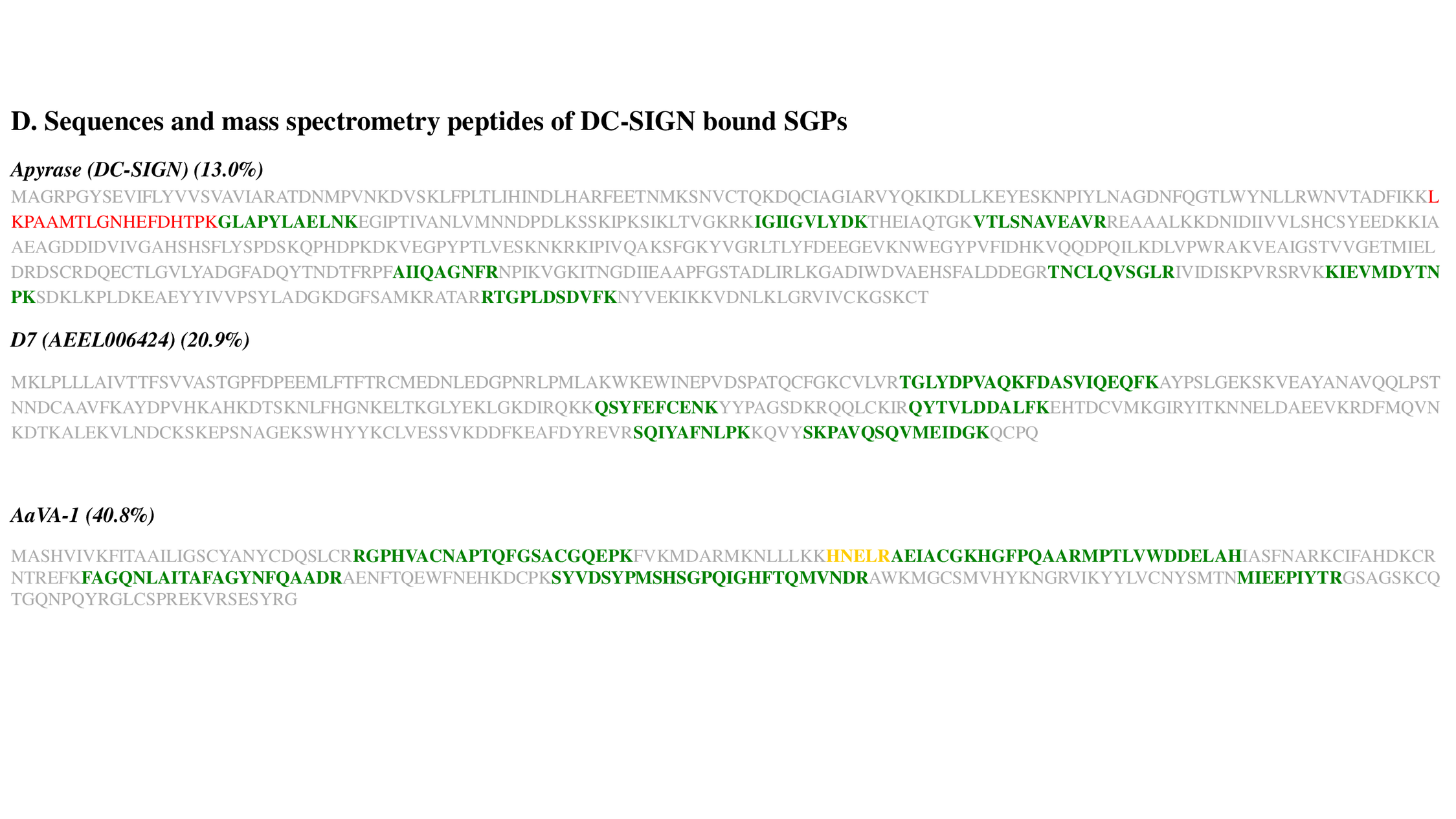

Supplement: S8 Fig — The individual SGPs associated with DC-SIGN human receptor protein and unique peptide coverages are provided. The green peptides represent positive reliable peptides of the proteins with high confidence making the overall percentage coverage indicated by each SGP while the red peptides represent very poor intensity and yellow represents low confidence intensity. The grey areas represent undetected regions forming majority of the peptides. (TIF) [file pntd.0010743.s008.tif]

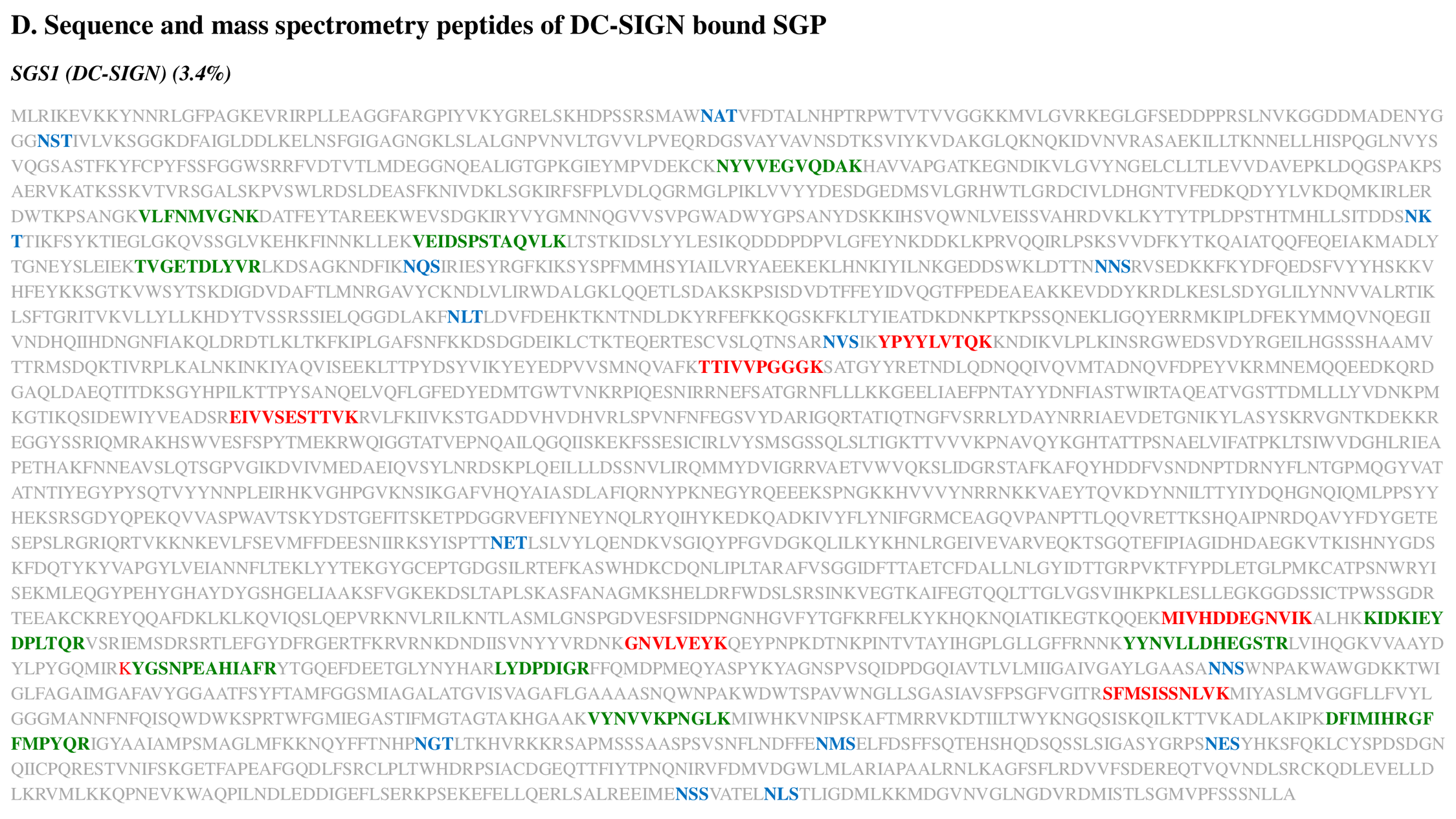

Supplement: S9 Fig — Sequence of the SGP associated with DC-SIGN human receptor protein and unique peptide coverages are provided. The green peptides represent positive reliable peptides of the proteins with high confidence making the overall percentage coverage indicated by each SGP while the red peptides represent very poor intensity and yellow represents low confidence intensity. The grey areas represent undetected regions forming majority of the peptides. Possible glycosylation/post-translational modification sites in SGS1 which could account for low peptide identification are indicated with boldened blue font (NXX), where XX represents two amino acid residues after the asparagine (N). (TIF) [file pntd.0010743.s009.tif]

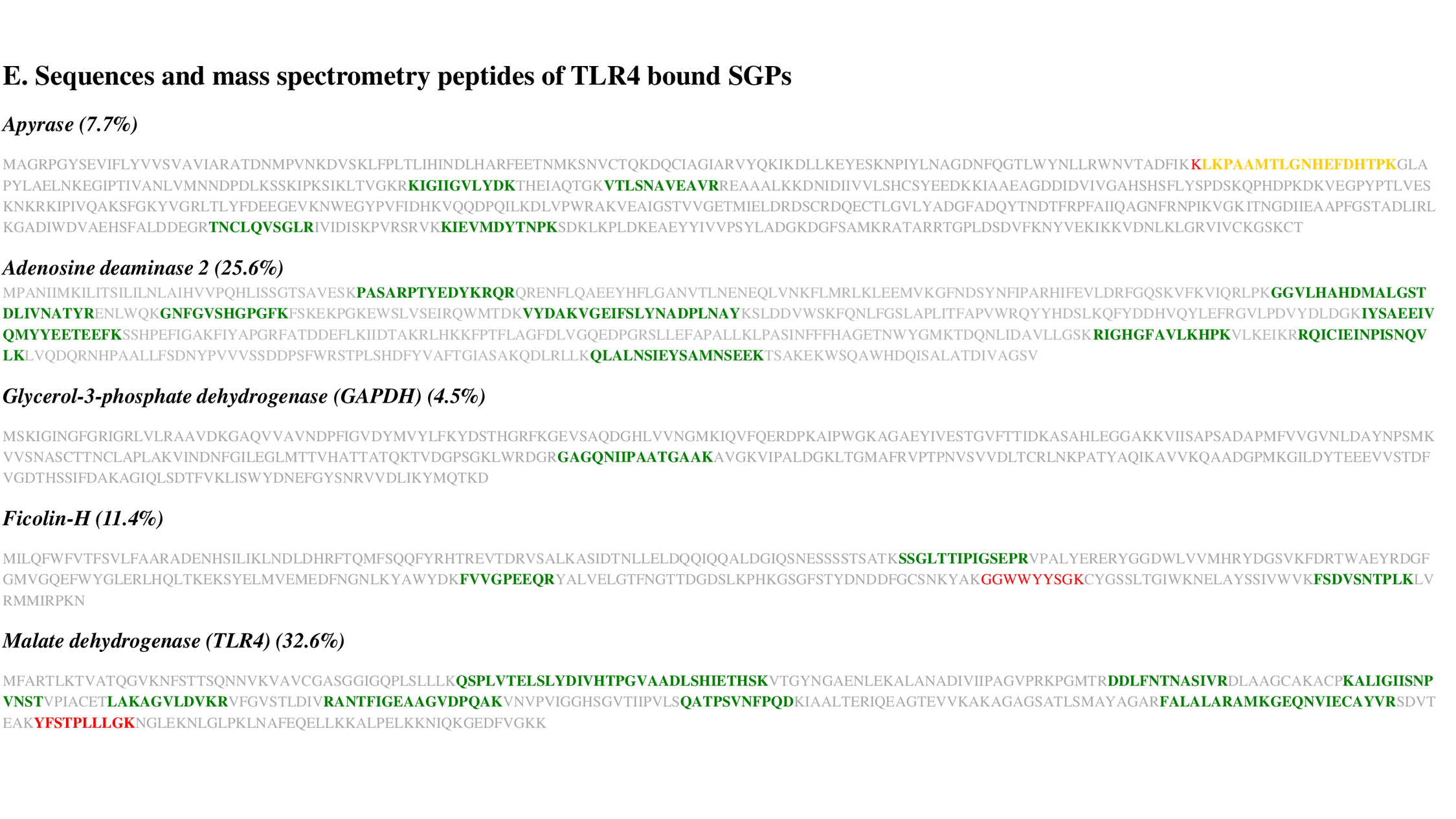

Supplement: S10 Fig — The individual SGPs associated with TLR4 human receptor protein and unique peptide coverages are provided. The green peptides represent positive reliable peptides of the proteins with high confidence making the overall percentage coverage indicated by each SGP while the red peptides represent very poor intensity and yellow represents low confidence intensity. The grey areas represent undetected regions forming majority of the peptides. (TIF) [file pntd.0010743.s010.tif]
